# A peptidyl-glucosamine derivative affects IKKα kinase activity in human chondrocytes

**DOI:** 10.1186/ar2920

**Published:** 2010-01-29

**Authors:** Anna Scotto d'Abusco, Laura Politi, Cesare Giordano, Roberto Scandurra

**Affiliations:** 1 Department of Biochemical Sciences, Sapienza University of Roma, P.le Aldo Moro, 5, 00185 Roma, Italy; 2 Institute of Biomolecular Chemistry, CNR, Sapienza University of Rome, P.le Aldo Moro, 5, 00185 Roma, Italy

## Abstract

**Introduction:**

Nuclear factor-κB (NF-κB) transcription factor regulates several cell signaling pathways, such as differentiation and inflammation, which are both altered in osteoarthritis. Inhibitor κB kinase (IKK)α and IKKβ are kinases involved in the activation of the NF-κB transcription factor. The aim of the present study was to determine the effects of glucosamine (GlcN), which is administered in the treatment of osteoarthritis, and of its 2-(N-Acetyl)-L-phenylalanylamido-2-deoxy-β-D-glucose (NAPA) derivative on IKK kinases and, consequently, on NF-κB activation in human chondrocytes.

**Methods:**

The human chondrosarcoma cell line HTB-94 and human primary chondrocytes were stimulated with tumor necrosis factor (TNF)α after pre-treatment with GlcN or NAPA. Gene mRNA expression level was evaluated by real-time PCR. Inhibitor κB protein (IκB)α phosphorylation and p65 nuclear re-localization were analyzed by Western blotting; IKKα nuclear re-localization was also investigated by immunocytochemistry and Western blotting. IKK kinase activity was studied by *in vitro *kinase assay.

**Results:**

After TNFα stimulation, the mRNA expression level of some of the genes under NF-κB control, such as *interleukin (IL)-6 *and *IL-8*, increased, while treatment with GlcN and NAPA reverted the effect. We investigated the possibility that GlcN and NAPA inhibit IKK kinase activity and found that NAPA inhibits the IKKα kinase activity, whereas GlcN does not. Interestingly, both GlcN and NAPA inhibit IKKα nuclear re-localization.

**Conclusions:**

Our results demonstrate that glucosamine and its peptidyl derivative can interfere with NF-κB signaling pathway by inhibiting IKKα activity in human chondrocytes. However, the mechanism of action of the two molecules is not completely overlapping. While NAPA can both specifically inhibit the IKKα kinase activity and IKKα nuclear re-localization, GlcN only acts on IKKα nuclear re-localization.

## Introduction

Osteoarthritis (OA), the most common rheumatic disease, is a major cause of disability. It is strongly associated with aging and its medical relevance is rising in the Western population given the increasing proportion of older people. This pathology is characterized by progressive destruction of the extracellular matrix (ECM), causing pain and disability in patients. OA is a non-curable disease and its pharmacological treatment is based mainly on analgesic agents or non-steroidal anti-inflammatory drugs (NSAIDs). Structure-modifying agents are also administered to OA patients, with the aim of preventing or delaying cartilage degradation by pharmacological treatment [1]. Several chondroprotective agents, such as glucosamine (GlcN), condroitin sulfate, diacerein and curcumin, have been studied [2-6]. To date, studies performed *in vivo *and *in vitro *on GlcN and condroitin sulfate have provided partially inconsistent results [7-11]. Since these agents are widely available and generally well tolerated and possess safer profiles compared with NSAIDs, it is important to understand their mechanism of action in detail.

We have previously studied GlcN and its N-acetyl phenylalanine derivative (NAPA) *in vivo*, in an animal model and *in vitro*, in primary chondrocytes and in an immortalized cell line. In the *in vivo *study, we found that both GlcN and NAPA were very effective in reducing cartilage changes induced in rabbit knee by intra-articular injection of vitamin A [12]. In the *in vitro *study, GlcN and NAPA were able to counteract the effects induced by inflammatory cytokines, tumor necrosis factor-alpha (TNFα) and interleukin (IL)-1β, both in human primary chondrocytes and in immortalized cell line lbvpa55 [13,14]. Interestingly, we found that GlcN inhibits matrix metalloproteinase production by inhibiting the phosphorylation of the mitogen-activated protein (MAP) kinases involved in the activation of activator protein-1 (AP-1) transcription factor complex [14]. NAPA showed the same behaviour (unpublished data). Furthermore, we found that several genes upregulated by TNFα are modulated by GlcN and NAPA [13]. Since these genes are under the control of nuclear factor-kappa-B (NF-κB) transcription factor, we decided to analyze their mechanism of action in the context of the NF-κB pathway.

NF-κB is a family of transcription factors that play an important role in the immune system and that can influence gene expression events with an impact on cell survival, differentiation and proliferation [15,16]. The mammalian NF-κB family consists of five related transcription factors: p50, p52, p65 (RelA), c-Rel and RelB. The established model of NF-κB action states that, in unstimulated cells, inhibitor κB proteins (IκBs) sequester the inactive transcription factor in the cytoplasm. Stimulatory events lead to IκB protein phosphorylation, ubiquitylation and subsequent degradation. The end result is the release of the cytoplasmic NF-κB complex, which moves into the nucleus, where it drives the expression of its target genes [15-17]. The kinase responsible for IκB phosphorylation is the inhibitor κB kinase (IKK) complex. Two components of the IKK complex, IKKα and IKKβ, are involved in the release of the NF-κB active form. Proinflammatory stimuli activate IKKβ, which is essential for IκBα degradation. In contrast, IKKα only rarely activates IκBα [18] but has been reported to activate the NF-κB pathway by working as a nucleosomal kinase [19,20] that stimulates a distinct class of genes [21]. Moreover, a differential role of IKKα and IKKβ in the physiology and progression of OA chondrocytes was recently reported, suggesting that the OA phenotype is more related to IKKα than to IKKβ [22].

The aim of the present study is to investigate whether GlcN and NAPA could affect the activation of IKKα and IKKβ in chondrocytes stimulated with the proinflammatory cytokine TNFα. We found that NAPA and, albeit to a lesser extent, GlcN inhibit the expression of genes under NF-κB control. We analyzed the effect of both molecules on IκBα phosphorylation and on p65 nuclear translocation. We also evaluated whether NAPA and GlcN could affect IKKα and IKKβ activation and IKKα nuclear translocation. To circumvent the limitations of human primary chondrocytes such as poor yield, low proliferation and inter-individual variability of donor samples, we conducted the study on the immortalized cell line HTB-94 (SW1353; American Type Culture Collection, Manassas, VA, USA). For confirmation, some experiments were also performed on human primary chondrocytes.

## Materials and methods

### Cell culture

The HTB-94 human chondrosarcoma cell line (SW1353) was purchased from American Type Culture Collection and was grown in Dulbecco's modified Eagle's medium (DMEM) (HyClone, Logan, UT, USA) supplemented with L-glutamine, penicillin/streptomycin (HyClone), plus 10% fetal bovine serum (FBS). Experiments were performed in DMEM containing 1% FBS. Human primary chondrocytes were isolated as previously described [14] from cartilage obtained from healthy donors. Full ethical consent was obtained from all donors, and the experiments were performed in accordance with Sapienza University of Roma ethics committee guidelines. Cells were used at first passage in DMEM containing 1% FBS.

### Cell treatment

The HTB-94 cell line has been previously shown to be a good model to study inflammatory pathways [23]. Cells were seeded in plates at the required density. Cells were left untreated (CTL) or treated with 10 ng/mL recombinant TNF-α (PeproTech EC Ltd., London, UK) or pre-treated for 2 hours with 5 and 10 mM GlcN (Sigma-Aldrich, St. Louis, MO, USA) or with 2-(N-Acetyl)-L-phenylalanylamido-2-deoxy-β-D-glucose (NAPA), synthesized as previously reported [24]. After pre-incubation, the cells were stimulated with 10 ng/mL TNF-α for the required time. Cells were analyzed by immunocytochemistry or harvested and processed for quantitative real-time polymerase chain reaction (Q-RT-PCR), for Western blot analysis and for immunoprecipitation.

### RNA extraction and reverse transcription

Total RNA was extracted using TRIZOL reagent (Invitrogen Corporation, Carlsbad, CA, USA) in accordance with the manufacturer's instructions. Briefly, a confluent 60-mm plate of HTB-94 or human primary chondrocytes was washed with phosphate-buffered saline (PBS) and homogenized in 1 mL of TRIZOL reagent. RNA was stored at -80°C until used. cDNA was synthesized from 1 μg of total RNA, using reverse transcriptase Improm II (Promega Corporation, Madison, WI, USA) in accordance with the manufacturer's instructions, and analyzed by Q-RT-PCR.

### Real-time polymerase chain reaction

Q-RT-PCR analysis was performed using an ABI Prism 7300 (Applied Biosystems, Foster City, CA, USA). Amplification was carried out with 50 ng of cDNA, in 96-well plates, using SYBR Green PCR Master mix (Applied Biosystems) in a volume of 25 μL. Each sample was analyzed in triplicate. PCR conditions were 94°C for 10 minutes followed by 40 cycles of 94°C for 15 seconds and 60°C for 1 minute. Primers were designed using Primer Express software (Applied Biosystems) and were synthesized by Primm (Milan, Italy). The following primers were used: IL-6 forward, 5'-TGGCCTGAAAAAGATGGATGCT-3'; IL-6 reverse, 5'-AACTCCAAAAGACCAGTGATGATTT-3' (NM_000600); IL-8 forward, 5'-AGATATTGCACGGGAGAATATACAAA-3'; IL-8 reverse, 5'-GCAAACCCATTCAATTCCTGAA-3' (NM_000584); IκBα forward, 5'-TGATCACCAACCAGCCAGAA-3'; IκBα reverse, 5'-TCTCGGAGCTCAGGATCACA-3' (NM_020529); ICAM-1 forward, 5'-GGTGACCGTGAATGTGCTC-3'; ICAM-1 reverse, 5'-GCCTGCAGTGCCCATTATG-3' (NM_000201.2); Mcp-1 forward 5'-CGCTCAGCCAGATGCAATC-3'; Mcp-1 reverse, 5'-GCACTGAGATCTTCCTATTGGTGAA-3' (NM_02982); glyceraldehyde-3-phosphate dehydrogenase (GAPDH) forward 5'-GGAGTCAACGGATTTGGTCGTA-3'; GAPDH reverse, 5'-GGCAACAATATCCACTTTACCAGAGT-3' (NM_02046).

The results were analyzed using the Sequence Detection Systems software (Applied Biosystems), which automatically recorded the threshold cycle (C_t_). Untreated cell sample (CTL) was used as calibrator. The fold change for CTL was 1.0. Target gene C_t _values were normalized against GAPDH. Data were analyzed using the 2^ΔΔCt ^method and expressed as fold change compared with CTL.

### Western blotting

Human primary chondrocytes, treated as described above, were washed with PBS and lysated by nuclear extract kit (Active Motif, Carlsbad, CA, USA) to separate the cytosolic from the nuclear extract in accordance with the manufacturer's instructions. Extracts were resolved on 10% SDS-PAGE. Gels were transferred to Hybond C membranes (GE Healthcare Europe, Milan, Italy) by electroblotting (Bio-Rad Laboratories, Inc., Hercules, CA, USA) and probed with specific antibodies in accordance with the manufacturer's instructions. Antibodies against IKKα and β-actin were purchased from Sigma-Aldrich, and antibodies against fibrillarin, p-IκBα and p65 were from Santa Cruz Biotechnology, Inc. (Santa Cruz, CA, USA). Where indicated, the intensity of bands was compared by densitometric analysis using ImageJ 1.41 (National Institutes of Health, Bethesda, MD, USA) and reported as fold change.

### Immunoprecipitation of the IKK complex

To immunoprecipitate the activated IKK complex, HTB-94 cells were treated with 10 ng/mL TNFα for 10 minutes, scraped and homogenized in lysis buffer pH 7.5 (50 mM TRIS-Cl, 100 mM NaCl, 1% NP40, 0.25% Na-deossycolate, 1 mM EDTA). Whole-cell lysate (200 μg) was incubated with anti-IKKα antibody (Sigma-Aldrich) at 4°C for 16 hours and next treated with protein A-Agarose beads (Santa Cruz Biotechnology, Inc.). After 2-hour incubation, the beads were extensively washed with lysis buffer and assayed in an *in vitro *kinase assay as detailed below.

### Kinase assay

To determine the effect of NAPA and GlcN on TNFα-induced IKK complex activation, we performed an immunocomplex kinase assay. Immunoprecipitated (IP)-IKK complex, recombinant IKKα (Invitrogen Corporation) and IKKβ (Invitrogen Corporation) were analyzed by kinase assay in a mixture containing 50 mM Tris-Cl pH 7.4, 100 mM NaCl, 10 μCi γ-^32^P-ATP (PerkinElmer Italia - Life and Analytical Sciences, Monza [Milan], Italy), 5 mM MgCl_2_, 1 mM DTT and 2 μg of substrate glutatione S-transferase (GST) IκBα (Santa Cruz Biotechnology, Inc.) in the presence or absence of different concentrations of GlcN or NAPA. Kinase assay was performed at 30°C for 30 minutes, and the reaction was stopped by boiling with SDS sample buffer (Sigma-Aldrich) for 5 minutes. Finally, the proteins were resolved on 10% SDS-PAGE and transferred to Hybond C membranes (GE Healthcare Europe) by electroblotting (Bio-Rad Laboratories, Inc.). Membrane was exposed to x-ray film to visualize the radioactive bands. To determine the total amounts of IKKα/β in each IP sample, the same membrane was probed with anti-IKKα antibody.

### Immunocytochemistry and confocal microscopy

IKKα nuclear re-localization was visualized by confocal microscopy. HTB-94 cells were untreated (CTL) or treated with 10 ng/mL TNFα and with GlcN or NAPA plus TNFα. After treatment, cells were fixed with 4% paraformaldehyde and permeabilized with 0.3% Triton X-100. After washing with PBS, the cells were incubated overnight at 4°C with monoclonal anti-IKKα (sc-7606; Santa Cruz Biotechnology, Inc.) (diluted 1:50), washed with PBS and incubated for 1 hour at room temperature with Alexa Fluor 488 goat anti-mouse antibody (Invitrogen Corporation) (diluted 1:300). Slides were washed, incubated with DAPI (diamidino-2-phenylindole) (Invitrogen Corporation) to visualize nuclei, mounted and analyzed with a Leica 2500 confocal microscopy (Leica Microsystems, Wetzlar, Germany).

### Assessment of cell viability

To detect potential cytotoxic effects of NAPA, the survival of the cells treated with this molecule was evaluated using MTT (3- [4,5-dimethylthiazol-2-yl]-2,5-di-phenyltetrazolium bromide)-based colorimetric assay (Sigma-Aldrich) in accordance with the manufacturer's instructions. Briefly, 1.5 × 10^4 ^cells per well were seeded in a 96-well plate in a volume of 150 μL. NAPA was added at concentrations of 1, 2.5, 5 and 10 mM. Fifteen microlitres of MTT, a soluble tetrazolium salt solution, was added to the well 24, 48 and 96 hours after treatment, and the plate was incubated for an additional 4 hours. Afterwards, the culture medium was removed and 150 μL of solvent solution was added to dissolve the MTT formazan crystals. Spectrophotometric absorbance was measured at a wavelength of 570 nm. The background at 690 nm was subtracted.

### Statistics

Each experiment was performed at least three times. The statistical significance of the differences between mean values was determined by a two-tailed *t *test; *P *value of not more than 0.05 was considered significant. When appropriate, results are expressed as the mean ± standard error of the mean.

## Results

### GlcN and NAPA prevent the overexpression of TNFα-stimulated genes

**Figure 1:**
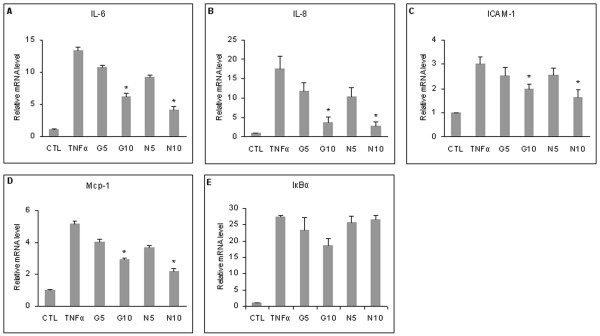
**Effect of glucosamine (GlcN) and NAPA on mRNA expression level in HTB-94 cells**. Cells were untreated (CTL), treated with tumor necrosis factor-alpha (TNFα) or pre-treated with 5 and 10 mM GlcN (G5 and G10) or NAPA (N5 and N10) and then stimulated with TNFα for 1 hour. The mRNA was extracted and analyzed by quantitative real-time polymerase chain reaction (Q-RT-PCR). The mRNA levels of IL-6, IL-8, ICAM-1, Mcp-1 and IκBα are shown in **(a)**, **(b)**, **(c)**, **(d) **and **(e)**, respectively. *P ≤ 0.05. Q-RT-PCR results are expressed as relative mRNA level. Results represent the mean ± standard error of the mean of data obtained by three independent experiments. NAPA, 2-(N-Acetyl)-L-phenylalanylamido-2-deoxy-β-D-glucose.

Previously, we found that both in immortalized cell line and in rabbit primary chondrocytes, GlcN and NAPA were able to counteract the TNFα upregulation of some genes, such as *TNFR*-1 and *TNFR*-2, *TRAF*-6 and *IGFBP*-6, whose transcription is under the control of NF-κB [12,13]. To explore whether GlcN and NAPA affect the NF-κB pathway in HTB-94 cells, we also analyzed the expression of other NF-κB-regulated genes. *IL*-6, *IL*-8, *ICAM*-1, *Mcp*-1 and *IκBα *mRNA expression levels were upregulated after 1-hour stimulation with TNFα. Two-hour pre-treatment with 10 mM of both molecules significantly reverted the stimulation of *IL*-6, *IL*-8, *ICAM*-1 and *Mcp*-1, whereas the effect on *IκBα *was negligible. The effect of GlcN and NAPA at a concentration of 5 mM was not significant (Figure 1). The same result was obtained in human primary chondrocytes (data not shown).

### GlcN and NAPA slightly affect IκBα phosphorylation and p65 nuclear migration

**Figure 2:**
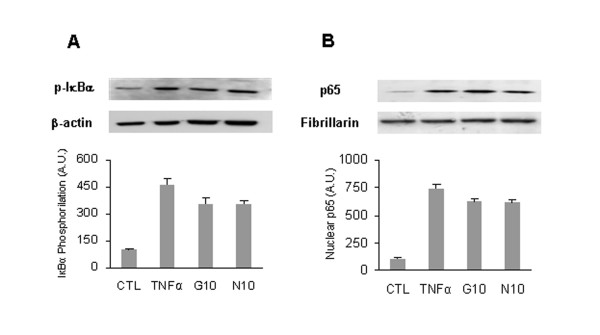
**Effect of glucosamine (GlcN) and NAPA on IκBα phosphorylation level and on p65 nuclear translocation**. HTB-94 cells were untreated (CTL), treated with tumor necrosis factor-alpha (TNFα) or pre-treated with 5 and 10 mM GlcN (G5 and G10) or NAPA (N5 and N10) and then stimulated with TNFα for 10 minutes. **(a) **Cytosolic extract probed with antibodies against phospho-IκBα (p-IκBα) and β-actin. **(b) **Nuclear extract probed with antibodies anti-p65 and fibrillarin. Band intensities were quantified as reported in Materials and methods. Results are expressed as fold changes with respect to control. The data are representative of three independent experiments. A.U., arbitrary units; NAPA, 2-(N-Acetyl)-L-phenylalanylamido-2-deoxy-β-D-glucose.

To determine whether GlcN and NAPA affected IκBα phosphorylation, we analyzed the latter protein by Western blot. IκBα was significantly phosphorylated in the cytosolic extract of cells stimulated with TNFα for 10 minutes. A 2-hour pre-treatment with GlcN and NAPA did not significantly inhibit IκBα phosphorylation (Figure 2a). Since a concentration of 5 mM of either molecules was ineffective in modulating gene expression, the experiments were performed with only 10 mM of both molecules. We investigated whether GlcN and NAPA inhibit the re-localization of the p65 subunit into the nucleus. Nuclear extract of cells treated for 10 minutes with TNFα showed that p65 was localized in the nucleus, an effect only very moderately inhibited by GlcN and NAPA, as expected given their minor effect on IκBα phosphorylation (Figure 2b). The same result was obtained on human primary chondrocytes (data not shown).

### NAPA affects the kinase activity of IKK complex

**Figure 3:**
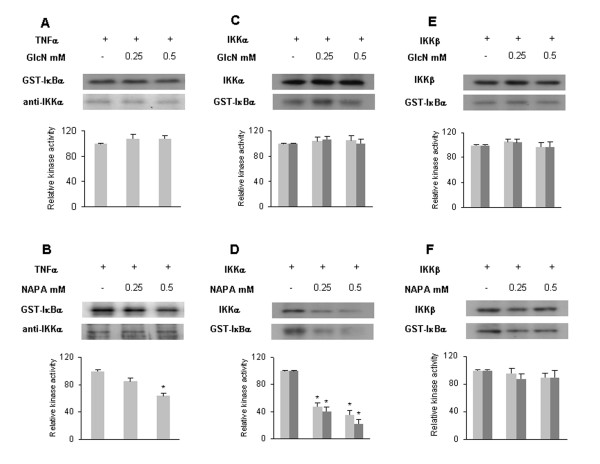
**Effect of glucosamine (GlcN) and NAPA on inhibitor κB kinase (IKK) kinase activity**. HTB-94 cells were stimulated for 10 minutes with tumor necrosis factor-alpha (TNFα), IKK complex was immunoprecipitated from whole-cell extract with an anti-IKKα antibody and an *in vitro *kinase assay was performed. **(a) **Kinase assay on recombinant glutatione S-transferase (GST)-IκBα in the absence (-) or presence of 0.25 and 0.5 mM GlcN. **(b) **Kinase assay on recombinant GST-IκBα in the absence (-) or presence of 0.25 and 0.5 mM NAPA. Normalization was obtained by Western blot analysis using anti-IKKα antibody. **(c) **IKKα kinase activity on itself, using IKKα recombinant protein, in the absence (-) or presence of 0.25 and 0.5 mM GlcN. **(d) **IKKα kinase activity on GST-IκB substrate in the absence (-) or presence of 0.25 and 0.5 mM NAPA. **(e) **IKKβ kinase activity on itself, using IKKβ recombinant protein, in the absence (-) or presence of 0.25 and 0.5 mM GlcN. **(f) **IKKβ kinase activity on GST-IκB substrate in the absence (-) or presence of 0.25 and 0.5 mM NAPA. Grey bars indicate auto-phosphorylation of IKKα or IKKβ as indicated, and dark grey bars show GST-IκBα phosphorylation. *P ≤ 0.05. Results are expressed as fold change with respect to control. NAPA, 2-(N-Acetyl)-L-phenylalanylamido-2-deoxy-β-D-glucose.

IκB phosphorylation is mediated by the IKK complex. To determine whether GlcN and NAPA interfere with the IKK kinase activity, we treated HTB-94 cells with TNFα and the IKK complex was immunoprecipitated using an anti-IKKα antibody from whole-cell extracts. The IP-IKK complex was analyzed in an *in vitro *kinase assay using a recombinant GST-IκBα protein as substrate both in the absence and in the presence of GlcN and NAPA. In the first case, activated IP-IKK was able to phosphorylate GST-IκBα, demonstrating that TNFα activates the IKK complex in our experimental model. GlcN was not able to inhibit GST-IκBα phosphorylation (Figure 3a), whereas NAPA inhibited GST-IκBα phosphorylation at a concentration of 0.5 mM (Figure 3b). To distinguish between the effects of IKKα and IKKβ, we analyzed the inhibition of IKK kinase activity on GST-IκBα by GlcN and NAPA, using recombinant IKKα and IKKβ molecules. GlcN was not able to inhibit either IKKα or IKKβ at either concentration used (0.25 and 0.5 mM) (Figure 3c, e). On the contrary, NAPA strongly inhibited the IKKα kinase activity on itself and on GST-IκBα at both concentrations (Figure 3d) but did not affect the IKKβ kinase activity on itself or on GST-Iκ Bα (Figure 3f). In these experiments, we were able to use lower concentrations of GlcN and NAPA (0.25 and 0.5 mM) than those used on intact cells (10 mM) because the molecules can directly interact with the kinases without needing to cross the cell membrane.

### GlcN and NAPA inhibit IKKα nuclear migration

**Figure 4:**
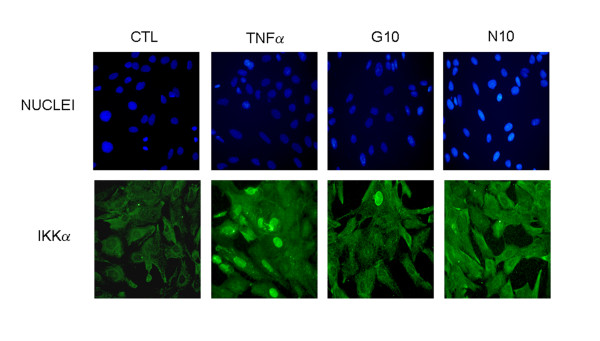
**Effect of glucosamine (GlcN) and NAPA on inhibitor κB kinase alpha (IKKα) nuclear translocation, analyzed by immunofluorescence**. HTB-94 cells were untreated (CTL), stimulated with tumor necrosis factor-alpha (TNFα) or pre-treated for 2 hours with 10 mM GlcN (G10) or NAPA (N10) and then stimulated with TNFα for 1 hour. Cells were then processed for indirect immunofluorescence and stained with anti-IKKα antibodies. Nuclei were stained with diamidino-2-phenylindole (DAPI). NAPA, 2-(N-Acetyl)-L-phenylalanylamido-2-deoxy-β-D-glucose.

**Figure 5:**
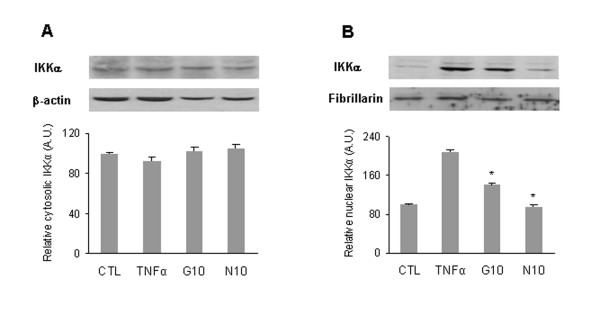
**Effect of glucosamine (GlcN) and NAPA on inhibitor κB kinase alpha (IKKα) nuclear translocation in human primary chondrocytes**. The analysis was performed by Western blot. Cells were untreated (CTL), treated with tumor necrosis factor-alpha (TNFα) or pre-treated with 10 mM GlcN (G10) or NAPA (N10) and then stimulated with TNFα for 1 hour. **(a) **Cytosolic extract probed with antibodies against IKKα and β-actin. **(b) **Nuclear extract probed with antibodies against IKKα and fibrillarin. *P ≤ 0.05. Results are expressed as fold change with respect to control. A.U., arbitrary units; NAPA, 2-(N-Acetyl)-L-phenylalanylamido-2-deoxy-β-D-glucose.

IKKβ activates the canonical NF-κB pathway by phosphorylation of IκBα, whereas IKKα is not required to phosphorylate IκBα, but it plays an important role by localizing into the nucleus of activated cells and inducing the transcription of NF-κB-dependent genes. To determine whether GlcN and NAPA could inhibit the IKKα nuclear translocation, we analyzed its subcellular localization by immunocytochemistry. Detection of IKKα revealed that this protein is mainly cytoplasmic in unstimulated cells, while it accumulates in the nucleus of cells stimulated with TNFα. Cells pre-treated with GlcN and NAPA and subsequently stimulated with TNFα showed a prevalent cytoplasmic IKKα localization (Figure 4). This result was confirmed in human primary chondrocytes by Western blot analysis in which both GlcN and NAPA were able to inhibit the re-localization of IKKα into nuclei (Figure 5a, b).

### NAPA inhibits nuclear IKKα kinase activity on histone H3

**Figure 6:**
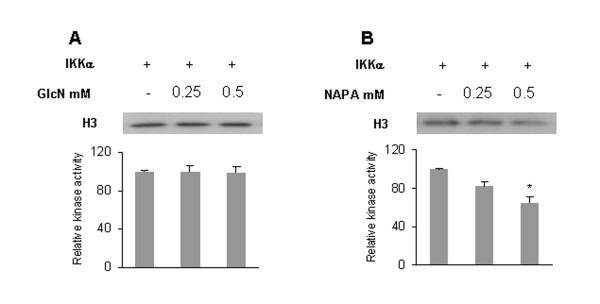
**Effect of glucosamine (GlcN) and NAPA on inhibitor κB kinase alpha (IKKα) kinase activity using recombinant histone H3 as substrate**. **(a) **IKKα kinase assay on recombinant histone H3 in the absence (-) or presence of 0.25 and 0.5 mM GlcN. **(b) **IKKα kinase assay on recombinant histone H3 in the absence (-) or presence of 0.25 and 0.5 mM NAPA. *P ≤ 0.05. Results are expressed as fold change with respect to control. NAPA, 2-(N-Acetyl)-L-phenylalanylamido-2-deoxy-β-D-glucose.

Several authors have shown that IKKα, after translocating into the nucleus, phosphorylates histone H3, thereby permitting the transcription of several genes under NF-κB control [19,20,25]. We investigated whether NAPA could inhibit the IKKα-dependent phosphorylation of histone H3 and indeed found that this is the case (Figure 6a). Interestingly, GlcN does not inhibit histone H3 phosphorylation (Figure 6b).

### NAPA does not interfere with chondrocyte viability

**Figure 7:**
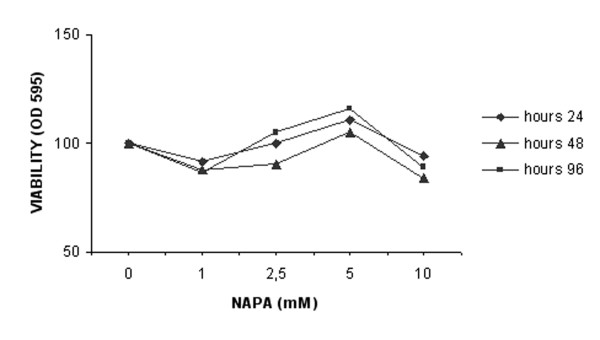
**Effect of NAPA on cell viability**. Cellular viability was assessed by MTT (3- [4,5-dimethylthiazol-2-yl]-2,5-di-phenyltetrazolium bromide) method after 24, 48 and 96 hours, with different concentrations of NAPA as indicated. NAPA, 2-(N-Acetyl)-L-phenylalanylamido-2-deoxy-β-D-glucose; OD, optical density.

To assess the potential cytotoxic effect of NAPA on human chondrocytes, we performed an MTT cell viability assay. The results show that NAPA does not affect cellular viability at any investigated concentrations or times (Figure 7).

## Discussion

The aim of the present study was to investigate the mechanism by which GlcN and its derivative NAPA affect the activation of the NF-κB transcription factor. NF-κB is an important regulator of the immune response but is also involved in a wide variety of stress responses and transcriptionally activates many genes with an important role in proliferation and matrix degradation.

Previously, we showed that the transcription of several genes under NF-κB control and stimulated by TNFα was modulated by both molecules [13]. Here, we show that other genes under NF-κB control, such as *IL*-6, *IL*-8, *ICAM*-1 and *Mcp*-1, are modulated as well in the HTB-94 chondrosarcoma cell line stimulated with TNFα. Proinflammatory cytokines can stimulate the NF-κB pathway by activating IKK complex, which is made up of IKKα, IKKβ and IKKγ/NEMO. The two IKKα and IKKβ subunits are homologous kinases, whereas NEMO is a regulator subunit [26].

In the canonical NF-κB pathway, IKKβ is sufficient for phosphorylation of IκBα, leading to its degradation and thereby allowing the translocation of p50/p65 in the nucleus [27]. On the other hand, after stimulation, IKKα itself migrates into the nucleus, where it stimulates gene transcription [19,28-30]. We tested the ability of GlcN and NAPA to inhibit IκBα phosphorylation and p65 nuclear translocation, finding that both molecules are weakly effective. Our results suggested that NF-κB-dependent gene modulation should be attributed to IKKα rather than to IKKβ. In an *in vitro *kinase assay, we analyzed the IP-IKK complex and found that GST-IκBα phosphorylation was mediated by the activated complex in the absence of NAPA or GlcN. This phosphorylation was inhibited by NAPA, while no effect of GlcN was detected. To dissect the roles of IKKα and IKKβ, we repeated the *in vitro *kinase assay using the individual recombinant kinases. Interestingly, we found that NAPA inhibited IKKα-mediated auto-phosphorylation and phosphorylation of GST-IκBα but had no effect on IKKβ. When IKKα migrates into the nucleus, it phosphorylates some substrates, derepressing the NF-κB target genes [31,32]. Among IKKα-phosphorylated substrates is the histone H3, which is subsequently acetylated [25]. This is a crucial step in modulating chromatin accessibility at NF-κB responsive promoter [19,20]. We found that NAPA can also inhibit H3 phosphorylation by IKKα, suggesting that this molecule is a specific inhibitor of IKKα kinase activity. GlcN was not able to inhibit either IKKα or IKKβ kinase activity.

We tested whether TNFα stimulates the migration of IKKα into the nucleus in chondrocytes as is the case in other cell types [19,20,25,26,33] and whether the effect could be inhibited by GlcN and NAPA. Indeed, TNFα stimulates a massive re-localization of IKKα into the nucleus in HTB-94 cell line and in human primary chondrocytes and both GlcN and NAPA are able to inhibit this migration. We could not detect an appreciable decrease of cytosolic IKKα in TNFα-stimulated cells, because of the high concentration of IKKα in this compartment. This result is in accordance with what was observed in other cell types [19,20,25,28,33]. The effectiveness of GlcN and NAPA in inhibiting IKKα nuclear migration explains the ability of these molecules to modulate the expression level of genes under NF-κB control.

Our data, in agreement with what was reported in [25], show that the absence of IKKα nuclear translocation and the inhibition of IKKα kinase activity modulate the transcription of genes under NF-κB control, regardless of the presence of p65, which is in the nucleus of GlcN- and NAPA-treated cells. Recently, a role for IKKα in accelerating nuclear clearance of p65 in macrophages was reported [34]. This could explain the nuclear accumulation of p65 that we observe in chondrocytes treated with both molecules: by inhibiting IKKα nuclear translocation, they might impair nuclear clearance of p65. Moreover, IKKα enhances promoter clearance in the nucleus [31,32] and recruits and mediates the phosphorylation of proteins [35], allowing binding of p65 to κB sequences. Consequently, the suppression of IKKα nuclear re-localization is expected to inhibit p65 binding.

In the IKKα kinase domain, a nuclear localization sequence (NLS), consisting of three lysines, Lys^236^-Lys^237^-Lys^238^, is present [33]. It has been shown that inactivation of NLS by site-direct mutagenesis prevents nuclear translocation but does not interfere with its kinase activity. To inhibit IKKα nuclear translocation, GlcN and NAPA should interfere with the NLS presumably by interacting with the lysine residues. This is consistent with their atomic structure since they are both stable pyranose hemiacetals in equilibrium with the open form in solution. The free aldehyde groups could react with the NH_2 _group of the lysine side chains. NAPA affects not only the nuclear translocation but also the kinase activity of IKKα. This is of relevance since inhibitors of enzymatic reactions are better suited for further optimization to increase their activity or pharmacokinetics properties.

It has been recently found that phenylethyl isothiocyanate shows anti-inflammatory properties acting via an attenuation of the NF-κB pathway in cancer cells [36,37]. Like NAPA, this molecule has an aromatic ring. This feature is shared by other molecules found to inhibit NF-κB activity, such as aspirine and salicylate [38], aminosalicylic acid [39] and curcumin (diferuloylmethane) [5,40]. Consistently, the structural difference between GlcN and its derivative is indeed the presence of an aromatic phenylalanine residue.

Cell activation by TNFα increases the transcription of the *IκBα *gene, which is under the control of the canonical NF-κB pathway activated by IKKβ [19,20,41]. GlcN and NAPA were not able to revert this increase, and this is consistent with the finding that both molecules inhibit IKKα but not IKKβ.

IKKα ablation was recently reported to show a broader range of effects on OA chondrocytes, such as enhanced ECM formation, due to the accumulation of collagen II fibers [22] and an increased chondrocyte proliferative capacity, a size reduction effect in undifferentiated chondrocytes and an enhanced survival rate of differentiated cells. It has been suggested that loss or inhibition of IKKα could ameliorate the degenerative aspects of OA chondrocytes, excessive ECM remodeling and increased cell death. Furthermore, since IKKα ablation increases the replicative potential and survival of OA chondrocytes, our results could be useful in the route of providing additional ways to attenuate OA progression. NAPA shows a specific effect on IKKα kinase activity and does not affect IKKβ kinase activity, and this makes it an interesting candidate for the treatment of the OA pathology.

## Conclusions

We have previously shown that GlcN and NAPA were both effective in restoring normal cartilage morphology in injured rabbit joints and that GlcN can inhibit AP-1 activation by inhibiting MAP kinase phosphorylation. Here, we show that GlcN and NAPA can also inhibit NF-κB activation and, specifically, that NAPA can inhibit IKKα kinase activity. Further studies are required to better understand the mechanism of action of the molecule and which other effects, besides mRNA transcription modulation, can be induced in cells. It has been suggested that IKKα inhibition could be a good strategy for OA treatment. Our results suggest that the NAPA peptidyl-GlcN derivative should be tested in association to glucosamine in the pharmacological treatment of OA.

## Abbreviations

AP-1: activator protein-1; C_t_: threshold cycle; CTL: untreated cell sample; DMEM: Dulbecco's modified Eagle's medium; ECM: extracellular matrix; FBS: fetal bovine serum; GAPDH: glyceraldehyde-3-phosphate dehydrogenase; GlcN: glucosamine; GST: glutatione S-transferase; IκB: inhibitor κB protein; IKK: inhibitor κB kinase; IL: interleukin; IP: immunoprecipitated; MAP: mitogen-activated protein; MTT: 3- [4,5-dimethylthiazol-2-yl]-2,5-di-phenyltetrazolium bromide; NAPA: 2-(N-Acetyl)-L-phenylalanylamido-2-deoxy-β-D-glucose; NF-κB: nuclear factor-kappa-B; NLS: nuclear localization sequence; NSAID: non-steroidal anti-inflammatory drug; OA: osteoarthritis; PBS: phosphate-buffered saline; Q-RT-PCR: quantitative real-time polymerase chain reaction; TNFα: tumor necrosis factor-alpha.

## Competing interests

The authors have filed a patent application based on the present work: patent pending number RM 2009 A000369.

## Authors' contributions

ASd'A conceived the design of the study, carried out the cell cultures, performed Q-RT-PCR, coordinated and trained others to perform the experiments, participated in statistical analysis and coordinated all phases of manuscript writing. CG carried out NAPA synthesis. LP and RS coordinated the laboratory work, participated in analyzing the data and helped to draft the manuscript. All authors read and approved the final manuscript.
